# Individualized Prediction of In-Plane Shear Stress–Strain Curves for Composites Using Early-Stage Digital Image Correlation Strain Fields

**DOI:** 10.3390/ma19122609

**Published:** 2026-06-17

**Authors:** Chongyu Ruan, Maowen Yao, Xiangyu Zhao, Zhisheng Yu, Guangwu Fang

**Affiliations:** 1 College of General Aviation and Flight, Nanjing University of Aeronautics and Astronautics, Liyang 213300, China; yuchongruan@163.com (C.R.); yao_maowen@163.com (M.Y.); zhao_xiangyu309@163.com (X.Z.); yuzhisheng@nuaa.edu.cn (Z.Y.); 2 Key Laboratory of Aero-Engine Thermal Environment and Structure, Ministry of Industry and Information Technology, College of Energy and Power Engineering, Nanjing University of Aeronautics and Astronautics, Nanjing 210016, China

**Keywords:** property scatter, CFRP, V-notched shear test, convolutional neural network, non-destructive evaluation

## Abstract

**Highlights:**

- Single early DIC strain map predicts full CFRP shear stress–strain curve.
- CNN maps 0.2% strain field to full curve with overall R^2^ = 0.945.
- Data augmentation and Dropout reduce RMSE by 40% vs. baseline.
- Individual-specific scatter captured (min R^2^ = 0.821, max = 0.992).

**Abstract:**

The in-plane shear performance of carbon fiber-reinforced polymer (CFRP) composites is critical for structural design but is challenged by significant property scatter. This study aims to achieve individualized prediction of the complete shear stress–strain curve for each composite specimen using only a single early-stage digital image correlation (DIC) strain field. Systematic in-plane shear tests were conducted on 45 laminated carbon fiber/epoxy specimens with synchronized full-field DIC data and macroscopic load–displacement records. A lightweight encoder–decoder convolutional neural network was developed, taking a single DIC strain contour map at 0.2% global strain as input and mapping it directly to the full-range stress–strain curve up to failure for that specific specimen. Data augmentation and Dropout regularization mitigated the small-sample challenge. The proposed model achieved strong predictive performance across the five-fold cross-validation yielded a mean R^2^ of 0.926 ± 0.022 and a mean RMSE of 6.37 ± 1.14 MPa for stress. Individual specimen predictions on the test set yielded an average R^2^ of 0.945, with a minimum of 0.821, confirming robust capability across scattered properties. Residual analysis elucidated error characteristics across deformation stages. This research provides a novel paradigm for non-destructive, early-stage individualized assessment of composite mechanical properties, with applications in structural health monitoring and probabilistic design.

## 1. Introduction

Carbon fiber-reinforced polymer (CFRP) composites have solidified their role as enabling materials in aerospace, automotive, and renewable energy sectors, primarily owing to their unparalleled specific strength and stiffness. Market analyses indicate that global CFRP demand has not only met but exceeded earlier projections, reaching approximately 285,000 metric tons by 2025 with a sustained compound annual growth rate of around 12.5% [1,2]. This expansion is largely fueled by the aggressive adoption in next-generation aircraft, offshore wind turbine blades exceeding 120 m, and the burgeoning hydrogen pressure vessel market. Within the spectrum of mechanical properties, in-plane shear behavior remains a critical determinant of structural integrity, particularly governing the failure mechanisms of multidirectional laminates under complex loading scenarios [3]. Consequently, despite advancements in manufacturing, the precise characterization and predictive modeling of CFRP in-plane shear response continue to pose substantial scientific and engineering challenges.

The difficulty primarily stems from two sources. First, the manufacturing process inevitably introduces microstructural variabilities, such as fiber misalignment, void distribution, and local resin richness, that cause significant scatter in mechanical properties, even among specimens cut from the same panel [4,5]. Slovikov and Lobanov [6] demonstrated that internal voids at a concentration of 5.3% reduced the shear strength of CFRP by 2.2%, while wrinkles altered both modulus and strength. Huang et al. [7] further showed that microstructural variabilities have an increasing impact on the nonlinear responses of unidirectional CFRP parts under progressive loading. Such property dispersion poses serious challenges for deterministic design approaches that rely on mean material properties with safety factors.

Second, conventional methods for obtaining full stress–strain curves are inherently destructive and specimen-specific. Standard in-plane shear tests, such as the V-notched rail shear method (ASTM D7078 [8]) and the ±45° tensile test (ASTM D3518 [9]), provide macroscopic load–displacement data that are subsequently converted into stress–strain relationships [10]. While these methods are well-established, each stress–strain curve corresponds to a specific specimen that is inevitably destroyed during testing. This one-to-one destructive mapping makes it impossible to obtain the complete stress–strain response of a given specimen without testing it to failure.

In recent years, digital image correlation (DIC) has emerged as a powerful non-contact technique for full-field deformation measurement, offering unprecedented insight into strain localization and damage progression in composite materials [11,12,13]. Merzkirch [14] systematically reviewed the application of DIC in V-notched specimen testing, demonstrating its capability to capture shear strain distributions across the gauge section with high spatial resolution. Dan et al. [15] further advanced DIC technology by introducing transformer-based end-to-end models for accurate displacement and strain field prediction, enabling robust measurement of high-frequency deformation features. These advancements have positioned DIC as an ideal tool for extracting early-stage strain field information that potentially carries signatures of the specimen’s unique microstructural state.

Concurrently, data-driven and machine learning methods have revolutionized the prediction of material mechanical behavior. Xu et al. [16] provided a comprehensive review of data-driven approaches for property prediction in fiber-reinforced composites, categorizing methods from physics-informed feature engineering to cross-scale modeling. Specific applications to stress–strain curve prediction have proliferated. Kim et al. [17] developed a deep neural network combined with principal component analysis to predict stress–strain curves of unidirectional composites from microstructural features. Ding et al. [18] integrated convolutional neural networks with a constitutive model to achieve generalizable stress–strain curve prediction across different material constituents. Pan et al. [19] applied multiple data-driven models to predict stress–strain behavior in defect-containing ceramic matrix composites. Wu et al. [20] extended the Deep Material Network framework for stochastic composite surrogates, demonstrating its ability to account for micro-structure variability. Notably, Xu et al. [21] developed Crack-Net, a deep learning framework that simultaneously predicts crack propagation and stress–strain curves in particulate composites, achieving good accuracy with R^2^ values above 0.999 for short-term predictions.

Despite these advances, a critical gap remains. Most existing data-driven approaches require either microstructural images as inputs [17,18], extensive finite-element simulation data [20,21], or a priori constitutive assumptions [18,22]. They are not designed to leverage the intrinsic property scatter observed in physical experiments as a source of information rather than noise. Furthermore, the potential of using early-stage full-field strain measurements, acquired non-destructively via DIC, to predict the complete, specimen-specific stress–strain curve up to failure has not been systematically explored.

Herein, the research proposes a novel data-driven paradigm that embraces rather than disregards the inherent variability in composite shear properties. The research proposes a lightweight encoder–decoder convolutional neural network that takes as input a single DIC strain contour map at a low strain level and directly maps it to the full-range stress–strain curve of that specific specimen. The model is trained and validated on a comprehensive dataset of 45 CFRP specimens subjected to systematic in-plane shear tests, with synchronized full-field strain and macroscopic load–displacement data. Through targeted data augmentation and Dropout regularization, the research proposes the small-sample-size challenge inherent in physical experimentation. The proposed framework offers a non-destructive, early-stage approach for individual-level mechanical property assessment, with potential implications for structural health monitoring, quality control, and probabilistic design of composite structures.

## 2. Materials and Methods

### 2.1. Materials and Specimen Preparation

**Figure 1:**
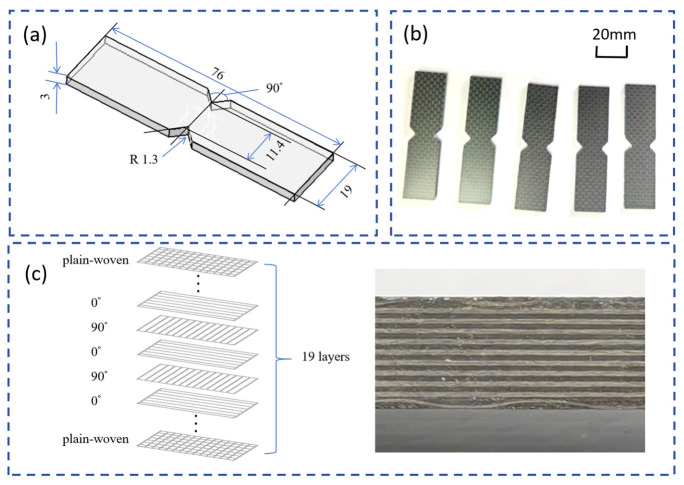
(**a**) Specimen geometry with dimensions, (**b**) photograph of the V-notched specimen, (**c**) schematic of the 19-ply layup.

The composite material used in this study was a carbon fiber-reinforced epoxy prepreg system (carbon fiber: Toray T700, Tokyo, Japan; epoxy matrix: commercial 350 K Cure system, Hexcel Corporation, Stamford, CT, USA). The prepreg was cured in an autoclave at 120 °C under 0.6 MPa pressure for 2 h, followed by a post-cure at 150 °C for 3 h, according to the manufacturer’s recommended cycle. The stacking sequence was designed as a symmetric layup. Specifically, the upper and lower surfaces consisted of plain-woven fabric layers, while the intermediate layers were arranged in alternating 0° and 90° unidirectional plies (Figure 1). A total of 19 plies were stacked, resulting in a nominal laminate thickness of 3.0 mm. The symmetric configuration ensured in-plane shear deformation without significant bending–shear coupling.

All specimens were machined into the V-notched rail shear configuration in accordance with ASTM D7078 [8]. The key geometric parameters are illustrated in Figure 1: overall length 76 mm, notch angle 90°, notch root radius R 1.3 mm, and central gauge width 19 mm. The V-notched ends were designed to fit the rail shear test fixture. Before testing, the specimen surfaces were lightly abraded with fine sandpaper (800 grit) and then coated with a matt white spray paint. A stochastic black speckle pattern was applied using an airbrush to enable high-resolution digital image correlation (DIC) measurements. A total of 45 specimens were fabricated from three independent plates (15 specimens per plate), where plate-to-plate variations in manufacturing tolerance and local material properties introduce batch-specific effects. To eliminate optimistic performance estimation caused by data leakage between training and test subsets, a strict partition constraint was enforced that no specimen from the same parent plate may appear in both the training set and test set of any single validation fold. The details can be found in Section 2.4.

### 2.2. In-Plane Shear Testing and Data Acquisition

**Figure 2:**
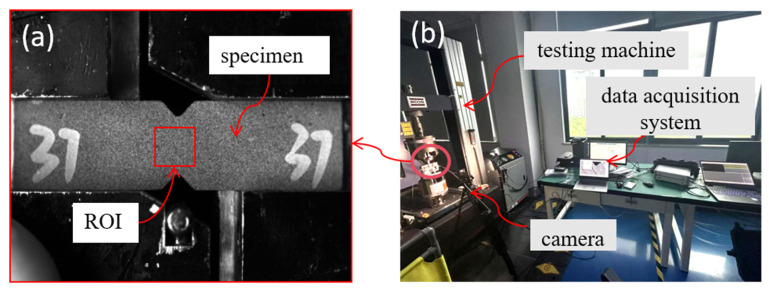
Schematic of the experimental setup for in-plane shear testing. (**a**) Enlarged view of the V-notched specimen mounted in the rail shear fixture, indicating the V-notch geometry, and the region of interest (ROI) for DIC strain measurement; (**b**) overall test system, including the universal testing machine, rail shear fixture, DIC camera, illumination source, and data acquisition computer.

In-plane shear tests were performed using a rail shear fixture mounted on a universal testing machine (Sansi, 50 kN capacity, Shenzhen, China). The test setup is schematically illustrated in Figure 2. A 5-megapixel monochrome digital image correlation (DIC) camera (5 MP monochrome, MV-CA050-10GM, Hikrobot, Hangzhou, China) was positioned perpendicular to the specimen surface at a working distance of approximately 400 mm, covering the entire V-notched gauge region. The DIC system was calibrated using a standard grid plate, yielding a spatial resolution of ~30 µm/pixel.

**Figure 3:**
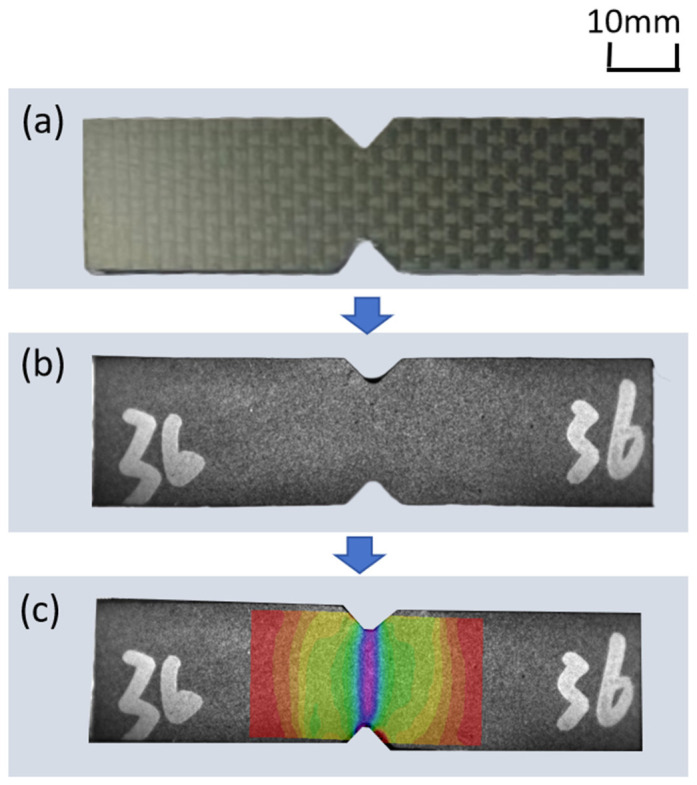
Specimen preparation and representative DIC strain field. (**a**) Bare V-notched shear specimen; (**b**) same specimen after application of white base coat and black stochastic speckle pattern; (**c**) representative shear strain contour map at 0.5% global strain, showing a highly uniform strain distribution in the central gauge region.

Before testing, each specimen was coated with a white base paint followed by a stochastic black speckle pattern (typical speckle size 3~5 pixels) to enable optimal DIC tracking. Figure 3a shows a bare specimen before painting, while Figure 3b presents the same specimen after speckle preparation. All tests were conducted under displacement control at a constant cross-head speed of 1 mm/min, following the quasi-static loading protocol recommended in ASTM D7078 [8]. The applied load and cross-head displacement were continuously recorded by the testing machine’s internal data acquisition system at a sampling rate of 10 Hz.

Simultaneously, DIC images were captured at a frame rate of 1 Hz (one image per second) throughout each test, from initial loading to final failure. The commercial software VIC-2D 2009.1.0 (Correlated Solutions, Inc., Irmo, SC, USA) was used for post-processing. A subset size of 29 × 29 pixels and a step size of 5 pixels were selected after convergence analysis. The strain fields were computed using a centered finite-difference scheme with a strain window size of 15 pixels. Figure 3c presents a representative contour map of the shear strain component *γ_xy_* at a global engineering shear strain of approximately 0.5%. Notably, a highly uniform shear strain distribution is observed across the central gauge region, confirming the effectiveness of the V-notched design in generating a pure and homogeneous in-plane shear state. This uniformity validates the use of a single strain contour map as the input feature for subsequent machine learning predictions. The DIC camera (1 Hz) and the testing machine load cell (10 Hz) were triggered by a common hardware trigger at the start of each test. Post-test, the load signal was down-sampled to 1 Hz using linear interpolation, and the resulting time-stamped load and strain data were aligned based on the trigger time stamp.

For each of the 45 valid specimens, the DIC-derived full-field strain data (at multiple time steps) were synchronized with the macroscopic load–displacement records, enabling the construction of complete experimental stress–strain curves up to failure. The engineering shear stress *τ* was calculated as follows:

(1) $$\tau =\frac{P}{A}$$

where *P* is the applied load and *A* is the specimen’s cross-sectional area within the gauge region (width 11.4 mm × nominal thickness 3.0 mm). The actual cross-sectional area of each specimen was measured individually using a digital vernier caliper (resolution 0.01 mm) before testing, taking the average of three measurements. The engineering shear strain *γ* was obtained from DIC by averaging the *γ_xy_* values over a rectangular region of interest (ROI) of 10 mm × 10 mm centered in the gauge area, as recommended by Merzkirch [14]. ROI extraction was performed automatically using a fixed pixel coordinate mask. The mask coordinates were calibrated on a reference image of the specimen fixture and applied consistently to all DIC strain maps. Manual intervention was not required after initial calibration.

### 2.3. Dataset Construction and Preprocessing

A total of 45 valid specimens were tested, each providing synchronized full-field DIC images and macroscopic load–displacement data. The goal of the dataset construction was to create paired input–output samples, where the input is a shear strain contour map extracted from the central gauge region at a low global strain level, and the output is the corresponding full-range engineering shear stress–strain curve of the same specimen.

From each test, the raw load–displacement data were first converted to engineering shear stress and strain. The strain data were extracted from the DIC analysis results (Excel files) and the stress was calculated as described in Section 2.2. For each specimen, the following preprocessing steps were applied:

- Invalid value removal: Data points with missing or obviously erroneous values (e.g., caused by DIC decorrelation or load cell spikes) were discarded.
- Monotonicity correction: Minor non-monotonicities in the stress–strain curve (due to measurement noise) were corrected by enforcing monotonic stress increase with respect to strain using a local smoothing algorithm.
- Moving average smoothing: To reduce high-frequency noise without distorting the overall trend, a moving average filter with a window size of 5 was applied to both stress and strain sequences.
- Strain normalization and resampling: To feed the curves into a convolutional neural network (CNN) as a fixed-length output vector, a fixed maximum strain value of 0.2 mm/mm was selected as the upper bound for strain normalization for all specimens. This value does not correspond to the specimen-specific failure strain; rather, it is a standardized normalization parameter applied uniformly across the entire dataset. Each stress–strain curve was truncated at 0.1 mm/mm (if a specimen failed below this strain, the curve was linearly extrapolated to 0.1 mm/mm). The strain axis was then normalized by dividing by 0.1 mm/mm, and the normalized curve was resampled to 128 equally spaced points using piecewise cubic Hermite interpolating polynomial (pchip) interpolation. Pchip was chosen because it preserves the monotonicity and shape of the original curve better than spline interpolation.

**Figure 4:**
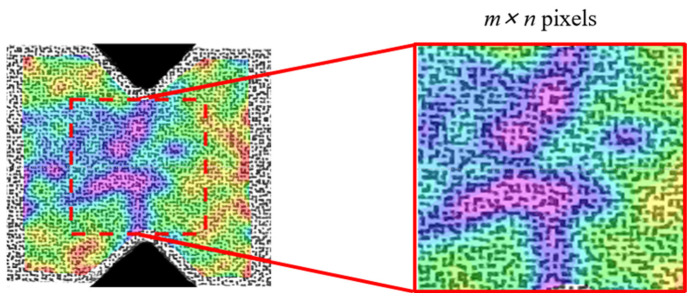
Extraction of the region of interest (ROI) from the full DIC shear strain field.

For each specimen, the DIC image captured at the lowest global strain level (targeting 0.2% engineering shear strain) was selected as the input. As shown in Figure 4, from the full DIC strain field, a fixed rectangular ROI of 10 mm × 10 mm was cropped, centered at the geometric middle of the V-notched gauge region. This ROI excludes edge effects and encompasses the area where the shear strain is most uniform.

**Figure 5:**
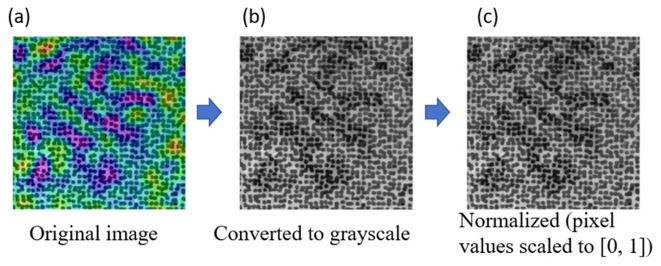
Image preprocessing pipeline for the strain contour map. (**a**) Original color contour map, (**b**) grayscale conversion and resizing to 224 × 224 pixels, (**c**) normalization to [0, 1] pixel values.

Figure 5 shows the step-by-step processing from the original color contour map to the final normalized grayscale image. The original color contour map of shear strain was converted to a grayscale image using standard luminance conversion. This step reduces data dimensionality while preserving the spatial distribution of strain gradients. The cropped grayscale image was resized to 224 × 224 pixels using bicubic interpolation. This size is compatible with lightweight CNN architectures (e.g., encoder–decoder networks) and provides sufficient spatial resolution for feature extraction. Then, the pixel values were normalized to the range [0, 1] by dividing by 255 (the maximum value of an 8-bit grayscale image).

After preprocessing, the dataset consisted of 45 input–output pairs. Each input is a 224 × 224 × 1 grayscale image (normalized strain contour at 0.2% global strain), and each output is a 128-dimensional vector representing the normalized stress values at 128 equally spaced normalized strain points.

### 2.4. Cross-Validation Strategy and Dataset Partition

All 45 specimens were fabricated from three independent plates (15 specimens per plate). To avoid optimistic performance estimates caused by data leakage between training and test sets while accounting for batch-to-batch variability, a stratified 5-fold cross-validation scheme was implemented.

- Specimens from each plate were randomly shuffled and evenly divided into 5 disjoint subgroups of 3 specimens each (15 ÷ 5 = 3, yielding zero imbalance).
- For the *k*-th fold (*k* = 1,…,5), the test set was composed of the *k*-th subgroup from all three plates, giving 9 test specimens (3 per plate). The training set consisted of the remaining 36 specimens (12 per plate).

This stratification guarantees that every fold contains representative samples from all three manufacturing batches, fully eliminating batch-effect leakage.

Two evaluation scenarios were conducted:

(1) ***CNN baseline without augmentation***: For each fold, the model was trained on the 36 original training specimens and tested on the 9 original test specimens. Performance metrics were averaged across the five folds.
(2) ***Enhanced CNN with augmentation***: To increase the effective sample size, each original strain contour map in the training set was augmented into three variants using mechanically admissible transformations: 180° rotation (equivalent under in-plane shear loading due to specimen symmetry) and Gaussian noise addition (σ = 0.01). From the resulting 108 augmented images (36 × 3), a validation set of 22 images (≈20%) was further separated, leaving 86 images for actual training. The test set retained the 9 original (non-augmented) specimens from the held-out subgroup. Augmentation was applied exclusively to the training subset of each fold; no augmentation was used for validation or testing.

Final metrics for both CNN configurations are reported as mean ± standard deviation across the five folds. After confirming hyperparameter stability via cross-validation, the final production model (enhanced CNN) was retrained on the full 45-specimen dataset with the same augmentation to maximize generalization.

### 2.5. Traditional Machine Learning Baselines for Comparison

To assess whether the complexity of the proposed CNN is justified, two non-deep learning baselines, linear regression and random forest, were implemented. Both models were trained on the same 36 original training samples per fold (without augmentation) and evaluated on the 9 original test samples, using the identical stratified 5-fold cross-validation scheme described in Section 2.4.

- ***Linear regression with PCA***: The input 224 × 224 strain contour map was flattened into a 50,176-dimensional vector. To avoid overfitting and reduce dimensionality, principal component analysis (PCA) was first applied to retain 95% of the variance, resulting in approximately 150–200 principal components. Linear regression was then performed on these PCA features to predict the 128-point stress vector. Each output dimension was modeled independently.
- ***Random forest with PCA***: Using the same PCA- reduced features as inputs, a random forest regressor with 100 trees was trained (other hyperparameters set to scikit-learn defaults). The output was again the 128-dimensional stress vector, with each dimension modeled independently.

Both baseline models were evaluated using the same performance metrics (RMSE and R^2^) as the CNN. The results are presented in Section 3.2.

### 2.6. Proposed CNN Architecture and Training

**Figure 6:**
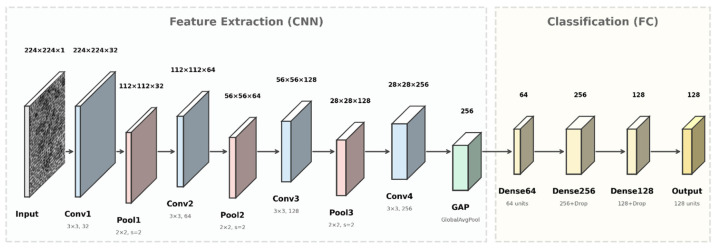
Network architecture of the proposed model.

A lightweight encoder–decoder style convolutional neural network (CNN) was developed to map the input strain contour map (224 × 224 grayscale image) directly to the output full-range stress–strain curve (128 normalized stress values). The overall architecture is illustrated in Figure 6.

The feature extraction backbone consists of four convolutional blocks followed by a global average pooling (GAP) layer. Each convolutional block uses 3 × 3 filters with ReLU activation and is followed by a 2 × 2 max-pooling (stride 2) for spatial downsampling. Specifically:

- Conv-Pool 1: 32 filters, output size 112 × 112 × 32;
- Conv-Pool 2: 64 filters, output size 56 × 56 × 64;
- Conv-Pool 3: 128 filters, output size 28 × 28 × 128;
- Conv-Pool 4: 256 filters, output size 14 × 14 × 256.

After the final pooling, a GAP layer reduces each feature map to a single scalar, producing a 256-dimensional feature vector. This vector passes through three dense layers:

- Dense 64: 64 units, ReLU activation;
- Dense 256: 256 units, ReLU activation, followed by Dropout (rate 0.5);
- Dense 128: 128 units, ReLU activation, followed by Dropout (rate 0.5).

The output layer consists of 128 units with linear activation, corresponding to the predicted shear stress values at the 128 normalized strain points.

The total number of trainable parameters is 1,255,176, making the model lightweight and suitable for small-sample training. Dropout is applied to prevent overfitting given the limited dataset size (45 specimens).

The dataset was split into training, validation, and test sets. The model was trained using the mean squared error (*MSE*) loss function between the predicted and ground-truth stress vectors:

(2) $$MSE=\frac{1}{N}\sum_{i=1}^{N}\sum_{j=1}^{128}{\left(\tau_{i,j}^{predicted}-\tau_{i,j}^{truth}\right)}^{2}$$

where *N* is the batch size, $\tau_{i,j}^{predicted}$ denotes the predicted stress at the *j*-th normalized strain point for specimen *i*, and $\tau_{i,j}^{truth}$ is the corresponding ground truth.

## 3. Results

### 3.1. Experimental Data Characterization

**Figure 7:**
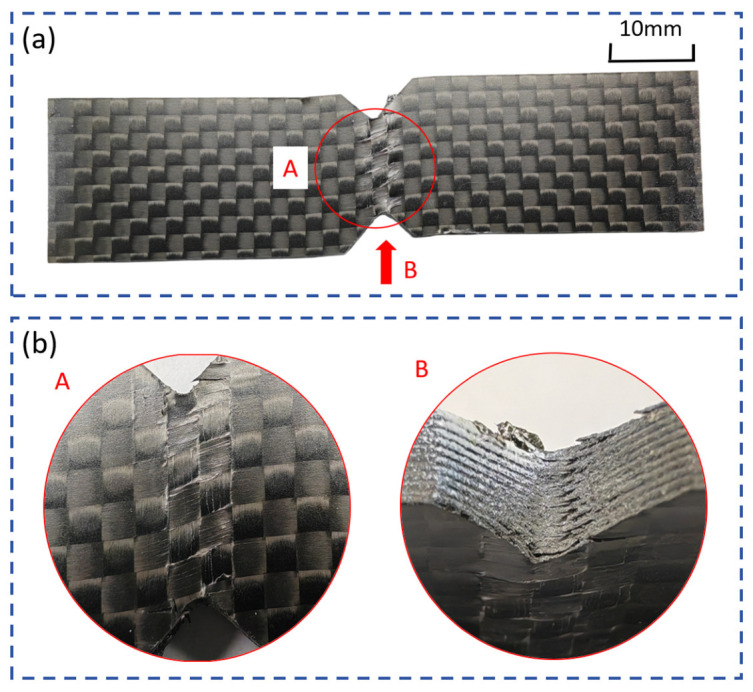
Fracture morphology of a representative shear specimen. (**a**) Overall view; (**b**) close-up views of regions A and B indicated in (**a**), revealing delamination details.

After in-plane shear testing, all specimens exhibited typical V-notch shear failure. Figure 7 shows a representative fractured specimen. The macroscopic image reveals crack propagation along the V-notched roots. The magnified views (front and side) clearly indicate pronounced interlaminar delamination near the notch tip, which is a characteristic failure mode in V-notched shear tests of multidirectional laminates. The delamination likely originates from the free edge effect and the high interlaminar shear stress concentration at the notch root.

**Figure 8:**
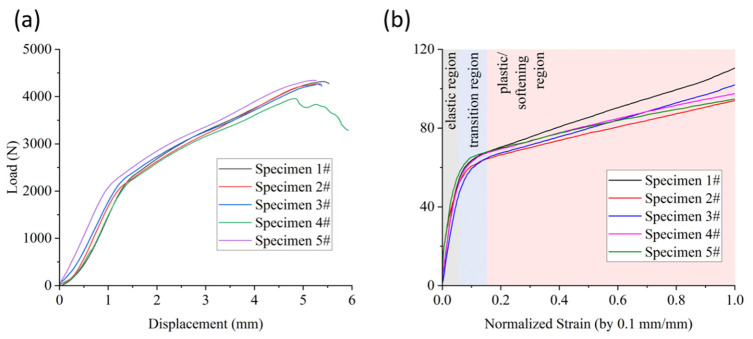
Experimental data characterization. (**a**) Load–displacement curves for 5 typical specimens; (**b**) corresponding engineering shear stress–strain curves showing clear nonlinearity and considerable inter-specimen scatter.

The macroscopic mechanical responses of typical specimens are presented in Figure 8. Panels (a) and (b) show the raw load–displacement curves and the converted engineering shear stress–strain curves, respectively. For consistency in curve normalization and model training, all stress–strain curves were truncated at a fixed shear strain of 0.1 mm/mm (indicated by the vertical dashed line in Figure 8b). This strain limit was selected as it remains within the early nonlinear range for most specimens and avoids the need to reach specimen-specific failure, thereby preserving the non-destructive nature of the approach.

As seen, all curves exhibit a distinctly nonlinear behavior. Typically, an initial linear elastic segment is followed by a transition zone (a gradual slope change), then a second nearly linear but softer segment, and finally a rapid failure with significant post-peak softening. This bilinear-like nonlinearity is typical for in-plane shear of cross-ply laminates, attributed to matrix microcracking and ply rotation. Furthermore, a considerable scatter among specimens is evident at all deformation stages—initial stiffness, transition strain, plateau stress, and ultimate failure. The coefficient of variation (CV) for the shear strength across the 45 specimens is approximately 8.7%, and the CV for the secant modulus (at 0.2% strain) is 7.2%. Such scatter reflects process-induced microstructural variabilities (e.g., ply waviness, void distribution, and local resin richness) that are inherent to autoclave-manufactured composites.

**Figure 9:**
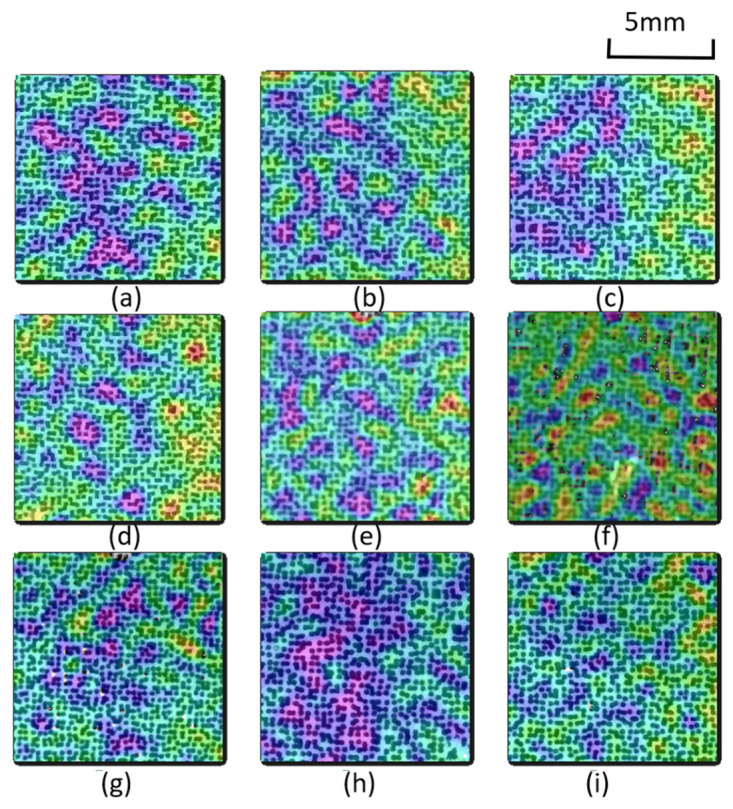
Shear strain contour maps at 0.2% global strain for 9 different specimens, showing inter-specimen variability. (**a**) Specimen 1; (**b**) Specimen 2; (**c**) Specimen 3; (**d**) Specimen 4; (**e**) Specimen 5; (**f**) Specimen 6; (**g**) Specimen 7; (**h**) Specimen 8; (**i**) Specimen 9.

Figure 9 displays the shear strain contour maps at a low global strain level (0.2%) for nine randomly selected specimens from the dataset. Remarkably, even at this early stage, far before any macroscopic nonlinearity or damage, the strain fields exhibit pronounced inter-specimen variability. Some specimens show a nearly uniform strain distribution across the gauge area, while others present local strain concentrations or asymmetric patterns. This early-stage strain heterogeneity likely originates from the same microstructural features that cause the scatter in the global stress–strain curves. In other words, the specimen-specific damage initiation sites (e.g., clusters of voids, fiber misalignment zones) induce localized strain concentrations that can be captured by DIC at very low load levels. These early-strain fingerprints plausibly govern the subsequent damage evolution and, ultimately, the full-range shear stress–strain response of each individual specimen.

Consequently, the observed correlation between the early-stage strain field heterogeneity and the final mechanical response dispersion validates the central hypothesis of this work: the initial DIC strain pattern encodes a *mechanical signature* that enables individualized prediction of the complete stress–strain curve. This finding motivates the use of the 0.2% strain contour map as the sole input to the deep learning model.

**Figure 10:**
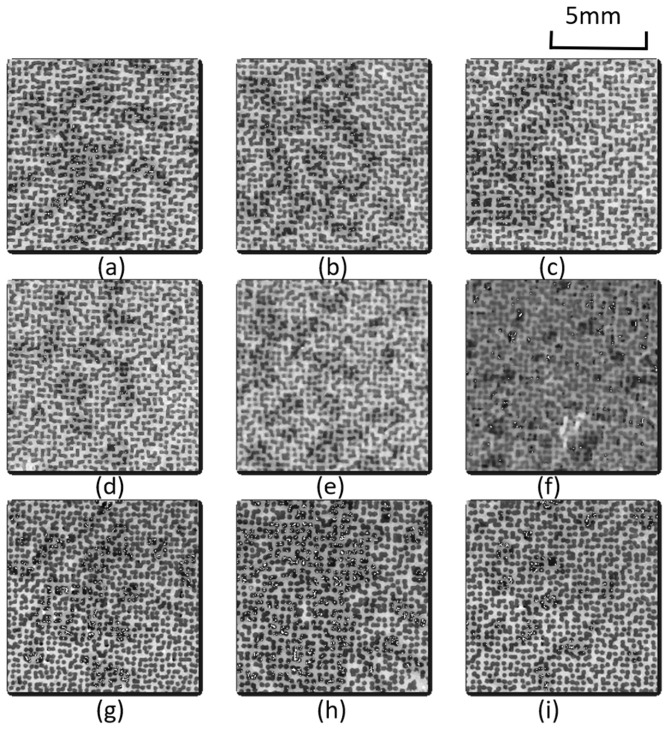
Preprocessed input strain contour maps (grayscale, normalized) for 9 test specimens. These images correspond to the same specimens as in Figure 9, after ROI extraction, resizing to 224 × 224 pixels, and pixel-wise normalization to [0, 1]. (**a**) Specimen 1; (**b**) Specimen 2; (**c**) Specimen 3; (**d**) Specimen 4; (**e**) Specimen 5; (**f**) Specimen 6; (**g**) Specimen 7; (**h**) Specimen 8; (**i**) Specimen 9.

Figure 10 presents nine representative preprocessed strain contour maps (grayscale, normalized to [0, 1]) from the test set. These images correspond to the same specimens shown in Figure 9 (original color contour maps) after the full preprocessing pipeline described in Section 2.3. The grayscale images effectively retain the spatial strain heterogeneity while reducing dimensionality.

### 3.2. Comparison of Baseline Models on the Original Dataset

Before evaluating the proposed CNN with data augmentation, we first compared the performance of three models on the original (non-augmented) dataset using the stratified 5-fold cross-validation described in Section 2.4. The results are summarized in Table 1.

The linear regression model combined with PCA achieved an RMSE of 11.75 MPa and an R^2^ of 0.741, indicating moderate predictive capability. However, its relatively high error and limited explained variance suggest that a linear mapping between the flattened strain field and the stress–strain curve is insufficient to capture the complex, nonlinear relationship.

Surprisingly, the random forest regressor with PCA features yielded an RMSE of 4.96 MPa but an extremely low R^2^ of 0.007. This near-zero R^2^ indicates that the random forest predictions are essentially no better than predicting the mean stress value for every sample, despite the apparently low RMSE. This counterintuitive result arises because the random forest model independently predicts each of the 128 stress points and fails to capture the shape and correlation structure of the stress–strain curve. In other words, the model produces outputs that have a small absolute error but are uncorrelated with the true stress values—highlighting the inadequacy of using RMSE alone as a performance metric for curve-shaped outputs.

The proposed CNN model (without augmentation) achieved an RMSE of 8.72 MPa and an R^2^ of 0.858, substantially outperforming both linear regression and random forest in terms of explained variance. Although its RMSE is higher than that of the random forest, the R^2^ value clearly demonstrates that the CNN captures the underlying functional relationship between the early-stage strain map and the entire stress–strain curve. This justifies the use of a deep learning architecture that can leverage spatial correlations in the strain field.

In summary, among the three baseline configurations on the original dataset, the CNN provides the most meaningful predictions, while the random forest fails to learn the curve structure and linear regression suffers from underfitting. These findings underscore the necessity of a spatially aware model such as a CNN for this individualized prediction task.

### 3.3. Cross-Validation Performance of the Enhanced CNN Model

A rigorous evaluation of the proposed data augmentation and Dropout regularization was conducted by comparing the enhanced CNN (with on-the-fly 180° rotation, contrast jitter, and Gaussian noise) against the baseline CNN (no augmentation, no Dropout). The identical stratified 5-fold cross-validation protocol described in Section 2.4 was used for both configurations. The per-fold and overall performance metrics are summarized in Table 2.

The enhanced CNN achieved an average R^2^ of 0.926 and an average RMSE of 6.37 MPa, markedly outperforming the baseline CNN (average R^2^ = 0.858, RMSE = 8.72 MPa). More importantly, substantially lower variability across folds was observed for the enhanced model: the standard deviation of R^2^ dropped from 0.072 (baseline) to 0.022 (enhanced), and the standard deviation of RMSE decreased from 2.43 MPa to 1.14 MPa. These results indicate that the regularization strategies not only improve predictive accuracy but also enhance stability and generalization across different training–test splits.

Fold 4 represents the most challenging case for the baseline model (R^2^ = 0.749, RMSE = 12.21 MPa). Notably, the enhanced CNN raised the R^2^ for this fold to 0.916 and reduced the RMSE to 7.07 MPa, demonstrating the effectiveness of augmentation in handling under-represented or harder-to-predict specimens. The best performance for the enhanced model occurred in Fold 5 (R^2^ = 0.961, RMSE = 4.51 MPa), which is comparable to the best baseline fold (Fold 2: R^2^ = 0.923).

**Figure 11:**
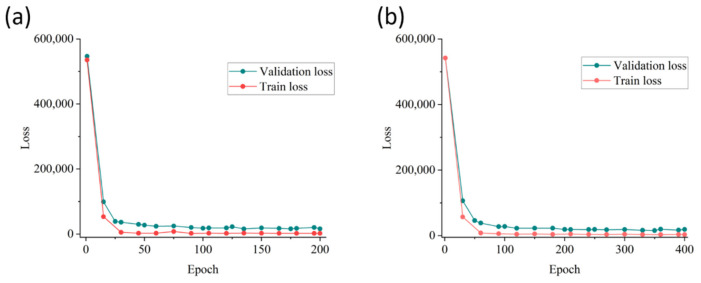
Training and validation loss curves for (**a**) the baseline CNN model (no augmentation, no Dropout) and (**b**) the enhanced CNN model (with augmentation and Dropout).

To further verify that the enhanced model does not suffer from overfitting, learning curves (training and validation loss versus epochs) were plotted for a representative fold. Figure 11 shows the loss evolution for the baseline CNN, and the enhanced CNN. Figure 11a presents the training and validation loss curves of the baseline CNN model over 200 epochs. Both losses decrease rapidly in the initial epochs and converge to relatively stable values, indicating that the model successfully learns from the training data. The training loss and validation loss exhibit similar convergence trends, suggesting that the baseline model achieves a reasonable balance between fitting the training data and generalizing to unseen data. Figure 11b illustrates the loss curves of the enhanced model over 400 epochs. Compared with the baseline model, the enhanced model demonstrates a comparable rapid decline in loss during the early training stage. Notably, the enhanced model maintains stable convergence over a longer training period, with both training and validation losses reaching lower final values. This indicates that the proposed enhancements contribute to improved optimization and potentially better generalization performance.

To assess the generalization capability of the enhanced model, both the baseline and the enhanced CNN were evaluated on an independent test set consisting of nine specimens that were completely held out from the cross-validation procedure (the test set from a single fold, following the stratification described in Section 2.4). The results are summarized in Table 3.

The enhanced model achieved an RMSE of 5.43 MPa and an R^2^ of 0.945, substantially outperforming the baseline model, which yielded an RMSE of 8.99 MPa and an R^2^ of 0.849. The improvements are consistent with the cross-validation results reported in Table 3. This independent test set validation confirms that the data augmentation and Dropout strategies effectively improve both predictive accuracy and generalization, without any overfitting to the specific fold partition.

### 3.4. Individual Prediction Performance: Best and Worst Cases

**Figure 12:**
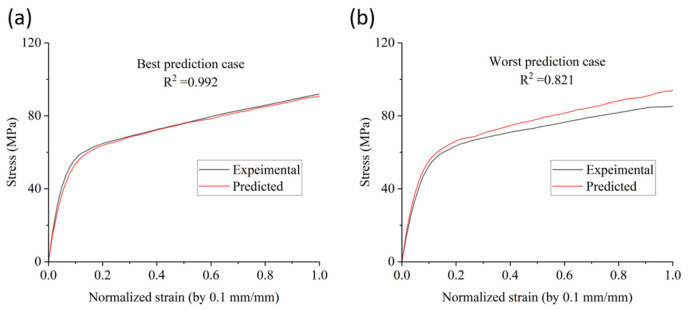
Predicted versus experimental stress–strain curves on the test set. (**a**) Best prediction; (**b**) worst prediction.

The best and worst individual predictions made by the enhanced CNN model on the test set are illustrated in Figure 12. The best prediction case (R^2^ = 0.992) is shown in Figure 12a, where an almost perfect overlap between the experimental and predicted stress–strain curves is observed across the entire strain range. This high-fidelity match indicates that the model successfully captured the specimen-specific nonlinear shear behavior, including the initial linear region, the transition zone, and the subsequent hardening or softening trend.

The worst prediction case (R^2^ = 0.821) is presented in Figure 12b. Here, the predicted curve generally follows the experimental trend but exhibits noticeable deviations, particularly in the intermediate strain range. The model slightly overestimates stress in this region, while the initial portions are more accurately captured. Despite being the poorest among the test set, an R^2^ of 0.821 still indicates a strong correlation, confirming the model’s robustness even for specimens with atypical mechanical responses. These results collectively demonstrate that the enhanced CNN provides reliable individualized stress–strain curve predictions across a wide range of shear behaviors.

The key advantage of the proposed approach lies in its ability to predict individual-specific mechanical behavior rather than merely reproducing an average response. Traditional mechanistic models or even many data-driven methods are trained to minimize mean squared error across the entire population, often yielding predictions that converge to the population mean for out-of-distribution samples. In contrast, our model uses the early-stage strain field, a fingerprint of the specimen’s unique micro-structure, to infer its entire future stress–strain trajectory. This individualized prediction capability is precisely what enables the high R^2^ values even for specimens whose behavior deviates substantially from the population average. Such a paradigm holds considerable promise for applications such as quality control (where one wants to assess the mechanical performance of a specific part non-destructively) and probabilistic structural design (where individual-level variability must be accounted for).

### 3.5. Residual Analysis

To further validate the predictive reliability of the proposed model, a residual analysis was conducted on the test set predictions. The residual for each stress prediction was defined as the difference between the experimental stress value and the model-predicted value:

(3) $$e=\tau_{i,j}^{predicted}-\tau_{i,j}^{truth}$$

**Figure 13:**
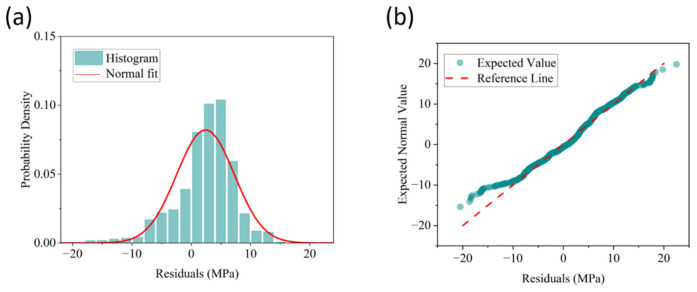
Residual analysis of model predictions on the test set. (**a**) Histogram of prediction residuals overlaid with a fitted normal distribution curve (red line); (**b**) Q-Q plot of residuals versus theoretical normal quantiles, with the diagonal red line representing perfect normality.

Figure 13a presents the histogram of residuals together with a fitted normal distribution curve. The residual distribution is approximately symmetric about zero, and the histogram bars closely follow the normal fit, indicating that the prediction errors are randomly distributed without systematic bias. Figure 13b shows the quantile–quantile (Q-Q) plot of the residuals against theoretical normal quantiles. The majority of points lie along or near the diagonal reference line, with only minor deviations observed at the extreme tails. This confirms that the residuals are approximately normally distributed. The normality of residuals supports the validity of the reported R^2^ and RMSE metrics and further demonstrates that the model produces unbiased predictions across the entire range of stress values. No evidence of heteroscedasticity or systematic over-/under-prediction was detected from either the histogram or the Q-Q plot.

This residual analysis, together with the high R^2^ values and low RMSE reported earlier, demonstrates that the proposed model not only achieves high accuracy but also produces statistically consistent and unbiased predictions across the entire range of shear stress values.

## 4. Discussion

### 4.1. Model Performance and Interpretability

The present study introduced a deep learning framework that uses an early-stage (0.2% global strain) DIC shear strain contour map as the sole input to predict the complete, specimen-specific stress–strain curve of CFRP composites under in-plane shear loading. The results demonstrate that this approach achieves strong predictive accuracy (R^2^ = 0.945, RMSE = 5.43 MPa) and, more importantly, captures the intrinsic inter-specimen variability with individual R^2^ values ranging from 0.813 to 0.994. This success stems from the fact that the early strain field acts as a *mechanical fingerprint* that encodes the specimen’s unique microstructural state, including fiber orientation, void distribution, and local resin richness, which governs subsequent damage initiation and propagation. Similar observations have been reported by Slovikov and Lobanov [6], who showed that void distributions directly affect shear strength, and by Xie et al. [5], who quantified how micro-defects induce property scatter in CFRP.

**Figure 14:**
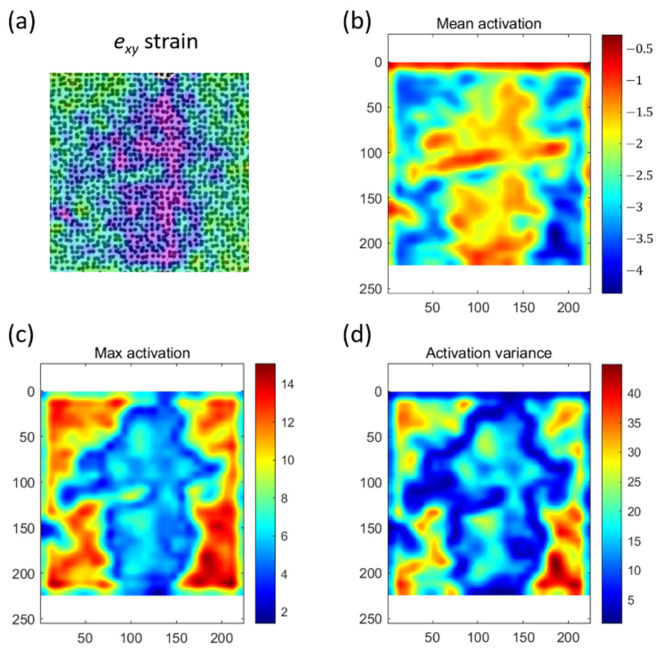
Feature map visualization of the last convolutional layer (Conv4). (**a**) Input DIC strain contour map; (**b**) mean activation map; (**c**) maximum activation map; (**d**) activation variance map.

To gain insight into what the CNN learns, the feature maps of the last convolutional layer (Conv4) were visualized for a representative input. Figure 14 presents the input strain contour map together with three different activation summaries. Figure 14a shows the original strain map, where a clear shear localization band develops along the V-notch region (purple/pink high-intensity areas). The mean activation map (Figure 14b) reveals that the model consistently focuses on the central gauge region, particularly the zones around the notch tips and the shear band, which are mechanically known as the sites of stress concentration and damage initiation [10,14]. This observation aligns with the Grad-CAM-based interpretability studies reported for composite damage prediction [23], where deep networks were shown to automatically identify physically meaningful regions. The maximum activation map (Figure 14c) exhibits high responses at image corners and outer edges, reflecting sparse strong reactions to boundary or intensity discontinuities rather than to the load-bearing area. The activation variance map (Figure 14d) shows similar corner-edge dominance, indicating that different channels converge on consistent feature extraction for the central mechanical region while producing divergent responses at edges. Collectively, these visualizations confirm that the model’s decision-making is physically interpretable: the primary attention is directed toward the V-notch tips and the developing shear band, which are the critical locations for shear failure.

The ability to predict individualized mechanical behavior has profound implications. Traditional deterministic characterization methods provide population-average properties, which often force designers to adopt conservative safety factors that penalize performance [1]. In contrast, the proposed data-driven paradigm enables non-destructive quality assessment of each manufactured part: by taking a single DIC image at a very low load level (well below the elastic limit), the entire shear stress–strain curve up to failure can be inferred without destroying the component. This capability is particularly attractive for structural health monitoring, in-process quality control during composite manufacturing, and probabilistic design frameworks that require realistic distributions of mechanical properties. Similar data-driven approaches have recently emerged in composite characterization; for example, Kim et al. [17] used PCA-aided deep neural networks to predict stress–strain curves from microstructural features, while Ding et al. [18] integrated CNNs with constitutive models for generalizable predictions. However, those approaches typically require explicit microstructural images or simulation data, whereas the present method directly leverages full-field experimental strain maps, which are more readily obtainable in practical testing scenarios.

### 4.2. Potential for Model Enhancement Using Multi-Time-Step Strain Fields

**Figure 15:**
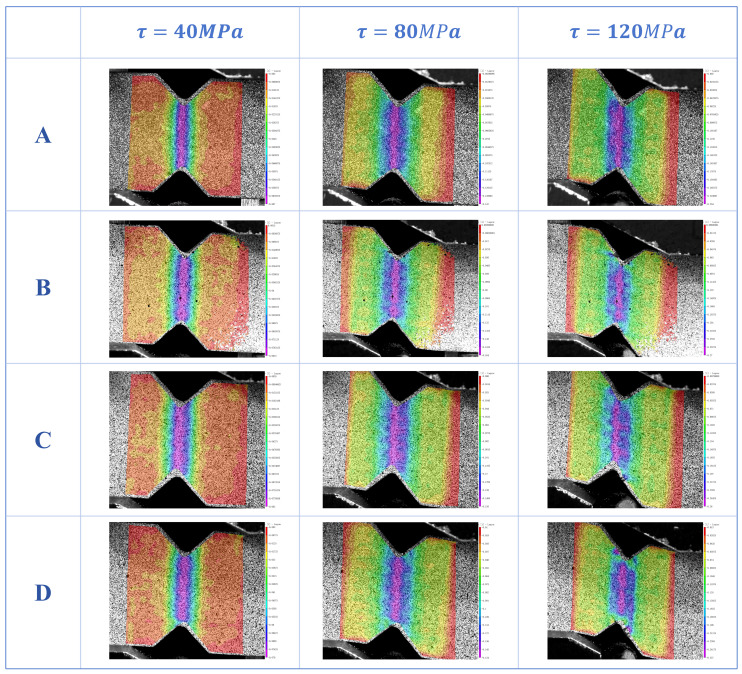
Evolution of shear strain contour maps at different global stress levels for four representative specimens (**A**–**D**). From left to right: early stage (≈40 MPa), intermediate stage (≈80 MPa), and near-failure stage (≈120 MPa). As stress increases, strain localization intensifies, and the pattern of damage evolution shows substantial inter-specimen variability.

The current model relies on a single strain field at a fixed low strain level. However, as shown in Figure 15, the evolution of the shear strain field is not static. At increasing global stress levels (e.g., 40 MPa, 80 MPa, and near failure), the strain field becomes progressively more heterogeneous, reflecting the emergence and coalescence of matrix microcracks, fiber–matrix debonding, and ultimately delamination. Figure 14 presents four representative specimens (A–D) at three stress levels. It is evident that the spatial patterns of strain localization vary considerably among specimens. Some develop a single intense shear band (Specimen B), while others exhibit a more diffuse, multi-band distribution (Specimen D). Moreover, the rate at which this heterogeneity grows differs across specimens, indicating that the damage evolution process itself is highly specimen-specific. These findings are consistent with those of Huang et al. [7] and Yang et al. [24], who demonstrated that microstructural heterogeneity leads to diverse damage progression paths.

The observation above suggests that the current model, which uses only the initial (0.2% strain) field, may not fully exploit the information contained in the later stages of damage progression. In fact, the initial strain field primarily captures the as-manufactured microstructural inhomogeneities (e.g., local stiffness variations). These initial defects indeed influence the entire subsequent failure process, which explains why the model already works well. However, damage nucleation and propagation are stochastic and can be further influenced by local stress redistributions that occur after microcracking. Therefore, including strain fields from multiple time points (e.g., at several strain levels before the onset of macroscopic damage) could provide a richer description of the material’s evolving internal state and potentially improve prediction accuracy, especially for specimens where damage initiation is not entirely predetermined by the initial state. This direction aligns with recent work by Xu et al. [21], who used time-series images to predict crack propagation and stress–strain curves in particulate composites.

### 4.3. Limitations and Future Perspectives

While the proposed framework achieves promising results, several limitations merit further discussion and point toward future research directions.

*Applicability to other laminate architectures.* The current model was trained and validated on a specific symmetric layup (woven surface layers with alternating 0°/90° unidirectional plies). Different stacking sequences (e.g., quasi-isotropic, angle-ply, or asymmetric laminates) produce different strain localization patterns and damage mechanisms [25]. Nevertheless, the methodology itself is generic; retraining or fine-tuning on a small set of DIC data from the target laminate would likely be sufficient. Transfer learning across layups should be investigated in future work.

*Sensitivity to DIC noise.* The quality of DIC measurements can be affected by factors such as speckle pattern quality, camera resolution, and ambient lighting. While the current model was trained on data acquired under controlled laboratory conditions, its robustness to varying noise levels in practical applications has not been systematically evaluated. It is anticipated that excessive noise could degrade prediction accuracy. Future work should include a dedicated sensitivity analysis using artificially added noise or real DIC data with different noise characteristics, and consider denoising preprocessing or noise-aware training strategies.

*Influence of ROI selection.* The region of interest (ROI) was manually fixed at a predetermined size and position centered on the gauge region. Although the model appeared to perform well with this choice, the sensitivity of predictions to the precise ROI boundaries has not been quantitatively assessed. Variations in ROI size or slight misalignments due to different test setups could potentially affect the input features. Future studies should investigate the robustness of the model to ROI selection, possibly by incorporating ROI-invariant features or by training with randomly cropped ROIs as a form of data augmentation.

*Robustness to different DIC acquisition settings.* The DIC parameters (e.g., subset size, step size, strain window) were kept constant during data acquisition and preprocessing. The model’s performance under different DIC settings (e.g., using a different camera, lens, or software with different post-processing algorithms) has not been validated. Domain shifts caused by these variations may reduce prediction accuracy [26]. Domain adaptation techniques or training with data from multiple DIC configurations could be explored to enhance generalizability.

*Transferability to other materials.* The current model was developed specifically for carbon/epoxy composites. Its direct transferability to other material systems (e.g., glass-fiber-reinforced polymers, natural-fiber composites, or metal sheets) is not guaranteed, as the mechanical behavior and strain evolution patterns differ. However, the proposed methodology is material-agnostic. Given a sufficient dataset for a new material, the same network architecture can be retrained. Transfer learning (fine-tuning with a smaller set of new material data) could accelerate adoption [27].

*Other limitations.* The limited dataset size (45 specimens) remains a challenge. Although data augmentation and Dropout effectively mitigated overfitting, a larger experimental database would allow training of deeper architectures or the use of more advanced methods such as generative adversarial networks or transfer learning from numerical simulations [16]. Furthermore, the current model outputs a normalized stress vector. Extending this framework to also predict failure strain or energy absorption would be valuable for toughness-critical applications. While this study focused on the V-notched shear test geometry, the methodology is generalizable to other loading configurations (e.g., ±45° tensile test, Iosipescu test) and other composite material systems. The key requirement is the availability of synchronized DIC strain fields and macroscopic stress–strain curves for a sufficiently diverse set of specimens. Future work should also explore uncertainty quantification frameworks such as the dual Bayesian model proposed by Li et al. [22].

In summary, the proposed early-stage DIC-based prediction framework is both effective and promising. The clear visual evidence of damage evolution scatter (Figure 15) and the interpretable feature maps (Figure 14) suggest that incorporating time-series strain maps could further elevate predictive performance while maintaining physical transparency. Nonetheless, the current model already delivers high-quality individualized predictions, opening new possibilities for non-destructive evaluation of composite shear properties.

## 5. Conclusions

This study aimed to achieve individualized prediction of the complete in-plane shear stress–strain curve for CFRP composites using only a single early-stage DIC strain contour map (0.2% global strain). A lightweight encoder–decoder CNN was developed and validated on 45 V-notched specimens with significant property scatter. The main findings are:

- The enhanced CNN model achieved strong predictive performance across the five-fold cross-validation yielded a mean R^2^ of 0.926 ± 0.022 and a mean RMSE of 6.37 ± 1.14 MPa for stress. Individual specimen predictions yielded an average R^2^ of 0.945 (minimum 0.821, maximum 0.992), confirming robust capability across scattered properties.
- Data augmentation and Dropout reduced RMSE by ≈40% compared to the baseline, effectively mitigating the small-sample challenge.
- Residual analysis showed unbiased, normally distributed errors, supporting statistical reliability.
- The key novelty is capturing individual-specific mechanical behavior using the early strain field as a “mechanical fingerprint” of microstructural variability.

In summary, this study provides a novel, efficient, and non-destructive paradigm for early-stage assessment of composite shear properties. The proposed framework holds promise for quality control, structural health monitoring, and probabilistic design of composite structures. Future work should explore the inclusion of time-series DIC data, transfer learning across different specimen geometries, and extension to other material systems and loading conditions.

## Figures and Tables

**Table 1:** Performance comparison of baseline models on the original dataset (no augmentation) using stratified 5-fold cross-validation.

Model Configuration	RMSE (MPa)	R^2^
Linear regression with PCA	11.75	0.741
Random forest with PCA	4.96	0.007
Proposed CNN model	8.72	0.858

**Table 2:** Five-fold cross-validation performance comparison between the baseline CNN (no augmentation) and the enhanced CNN (with augmentation and Dropout).

Statistic	Baseline CNN Model		Enhanced CNN Model	
	R^2^	RMSE (MPa)	R^2^	RMSE (MPa)
Fold 1	0.857	9.08	0.925	6.59
Fold 2	0.923	6.42	0.928	6.21
Fold 3	0.840	9.49	0.901	7.45
Fold 4	0.749	12.21	0.916	7.07
Fold 5	0.922	6.38	0.961	4.51
Average	0.858	8.72	0.926	6.37
Standard deviation	0.072	2.43	0.022	1.14

**Table 3:** Performance comparison on the independent test set (9 specimens) for the baseline and enhanced CNN models.

Model Configuration	RMSE (MPa)	R^2^
Baseline CNN model (no augmentation, no Dropout)	8.99	0.849
Enhanced CNN model (augmentation + Dropout)	5.43	0.945

## Data Availability

The original contributions presented in the study are included in the article. Further inquiries can be directed to the corresponding author.

## References

[1] Zhang J., Lin G., Vaidya U., Wang H. (2023). Past, present and future prospective of global carbon fibre composite developments and applications. Compos. Part B Eng..

[2] Luo Y., Shi Z., Qiao S., Tong A., Liao X., Zhang T., Bai J., Xu C., Xiong X., Chen F. (2024). Advances in nanomaterials as exceptional fillers to reinforce carbon fiber-reinforced polymers composites and their emerging applications. Polym. Compos..

[3] De B., Bera M., Bhattacharjee D., Ray B.C., Mukherjee S. (2024). A comprehensive review on fiber-reinforced polymer composites: Raw materials to applications, recycling, and waste management. Prog. Mater. Sci..

[4] Lobanov D.S., Slovikov S.V., Lunegova E.M. (2023). Influence of internal technological defects on the mechanical properties of structural CFRP. Fract. Struct. Integr..

[5] Xie C., Zhao Z., Sun L., Wang J., Jiang J., Li Y. (2025). Uncertainty analysis of the influence of micro-defects and delamination on the mechanical properties of CFRP. Compos. Struct..

[6] Slovikov S.V., Lobanov D.S. (2026). Experimental investigation of the influence of internal defects (voids, wrinkles) on the shear properties of CFRP. Fract. Struct. Integr..

[7] Huang T., Gao J., Sun Q., Zeng D., Su X., Liu W.K., Chen W. (2021). Stochastic nonlinear analysis of unidirectional fiber composites using image-based microstructural uncertainty quantification. Compos. Struct..

[8] (2020). Standard Test Method for Shear Properties of Composite Materials by V-Notched Rail Shear Method.

[9] (2018). Standard Test Method for In-Plane Shear Response of Polymer Matrix Composite Materials by Tensile Test of a ±45° Laminate.

[10] Adams D.O., Moriarty J.M., Gallegos A.M., Adams D.F. (2007). The V-notched rail shear test. J. Compos. Mater..

[11] Sutton M.A., Orteu J.J., Schreier H.W. (2009). Image Correlation for Shape, Motion and Deformation Measurements: Basic Concepts, Theory and Applications.

[12] Guseinov K., Kudryavtsev O., Bezmelnitsyn A., Sapozhnikov S. (2022). Determination of interlaminar shear properties of fibre-reinforced composites under biaxial loading: A new experimental approach. Polymers.

[13] Zhang Y., Han Q., Wen B. (2025). Phase-augmented digital image correlation for high-accuracy deformation measurement: Theory, validation, and application to constitutive law learning. J. Mech. Phys. Solids.

[14] Merzkirch M. (2022). V-notched specimen testing. Mechanical Characterization Using Digital Image Correlation: Advanced Fibrous Composite Laminates.

[15] Dan X., Guo H., Hu Y., Wang Y. (2025). Transformer-enhanced end-to-end models for accurate displacement and strain fields in digital image correlation. Opt. Express.

[16] Xu F., Liu L., Zhang C., Zhu J., Zhang W., Dong H., Huang H., Gao M., Yu X. (2025). Data-driven prediction of properties in fiber-reinforced composites. Sci. Technol. Rev..

[17] Kim D.W., Go M.S., Lim J.H., Lee S. (2023). Data-driven stress and strain curves of unidirectional composites by deep neural networks with principal component analysis and selective-data augmentation. Compos. Struct..

[18] Ding Z., Attar H.R., Wang H., Liu H., Li N. (2024). Integrating convolutional neural network and constitutive model for rapid prediction of stress-strain curves in fibre reinforced polymers: A generalisable approach. Mater. Des..

[19] Pan Y., Liu X., Ye W., Jin E., Xin J., Yao J. (2025). Data-driven prediction of stress–strain behavior in defect-containing Cf/SiBCN ceramic matrix composites: Accounting for multi-factor influences. J. Mater. Sci..

[20] Wu L., Noels L. Stochastic Deep Material Networks as efficient surrogates for composites & Deep Material Networks performance for damaging processes. Proceedings of the 8th International Conference on Computational Modelling of Fracture and Failure of Materials and Structures (CFRAC 2025).

[21] Xu H., Fan W., Ruan L., Shi R., Taylor A.C., Zhang D. (2025). Crack-Net: A deep learning approach to predict crack propagation and stress–strain curves in particulate composites. Engineering.

[22] Li T., Chen Z., Zhang Z., Wei Z., Zhong G.J., Li Z.M., Liu H. (2025). Predicting stress–strain curve with confidence: Balance between data minimization and uncertainty quantification by a dual Bayesian model. Polymers.

[23] Selvaraju R.R., Cogswell M., Das A., Vedantam R., Parikh D., Batra D. (2017). Grad-CAM: Visual explanations from deep networks via gradient-based localization. Proceedings of the IEEE International Conference on Computer Vision (ICCV), Venice, Italy, 22–29 October 2017.

[24] Yang D., Liu Y., Sun S., Wang Y. (2022). A deep learning framework for damage diagnosis of composite laminates using strain field. Compos. Struct..

[25] Pierron F., Grédiac M. (2012). The Virtual Fields Method: Extracting Constitutive Mechanical Parameters from Full-Field Deformation Measurements.

[26] Lecompte D., Smits A., Bossuyt S., Sol H., Vantomme J., Van Hemelrijck D., Habraken A.M. (2006). Quality assessment of speckle patterns for digital image correlation. Opt. Lasers Eng..

[27] Chen Z., Wang C., Wu J., Deng C., Wang Y. (2023). Deep convolutional transfer learning-based structural damage detection with domain adaptation. Appl. Intell..

